# Hand and Wrist Involvement in Seropositive Rheumatoid Arthritis, Seronegative Rheumatoid Arthritis, and Psoriatic Arthritis—The Value of Classic Radiography

**DOI:** 10.3390/jcm12072622

**Published:** 2023-03-30

**Authors:** Ewa Żelnio, Mihra Taljanovic, Małgorzata Mańczak, Iwona Sudoł-Szopińska

**Affiliations:** 1 Department of Radiology, National Institute of Geriatrics, Rheumatology and Rehabilitation, 02-637 Warsaw, Poland; 2 Department of Radiology, University of New Mexico, MSC08 4720, Albuquerque, NM 87106, USA; mihrat@radiology.arizona.edu; 3 Departments of Medical Imaging and Orthopaedic Surgery, University of Arizona, 1501 N. Campbell, Tucson, AZ 85724, USA; 4 Department of Gerontology, Public Health and Didactics, National Institute of Geriatrics, Rheumatology and Rehabilitation, 02-637 Warsaw, Poland

**Keywords:** rheumatoid hand, psoriatic arthritis, seronegative rheumatoid arthritis, seropositive rheumatoid arthritis, hand radiograph

## Abstract

The hand and wrist are among the most common anatomical areas involved in rheumatic diseases, especially seropositive and seronegative rheumatoid arthritis (RA) and psoriatic arthritis (PsA). The purpose of this study was to identify the most differentiating radiographic characteristics of PsA, seropositive RA, and seronegative RA, particularly in the early stages. A retrospective analysis of radiographic hand findings was performed on 180 seropositive RA patients (29 males, 151 females, mean age at the point of acquisition of the analyzed radiograph of 53.4 y/o, SD 12.6), 154 PsA patients (45 males, 109 females, age median of 48.1 y/o, SD 12.4), and 36 seronegative RA patients (4 males, 32 females, age median of 53.1 y/o, SD 17.1) acquired during the period 2005–2020. Posterior–anterior and Nørgaard views were analyzed in all patients. The radiographs were evaluated for three radiographic findings: type of symmetry (asymmetric/bilateral/changes in corresponding joint compartments/‘mirror-image’ symmetry), anatomic location (e.g., wrist, metacarpophalangeal (MCP), proximal interphalangeal (PIP), distal interphalangeal (DIP) joints), and type of lesions (e.g., juxta-articular osteoporosis, bone cysts, erosions, proliferative bone changes). The study showed that symmetric distribution of lesions defined as ‘lesions present in corresponding compartments’ was more suggestive of seropositive or seronegative RA than PsA. Lesions affecting the PIP joints, wrist, or styloid process of the radius; juxta-articular osteoporosis, joint space narrowing, joint subluxations, or dislocations were more common in patients with seropositive RA than in those with PsA, whereas DIP joints’ involvement and proliferative bone changes were more likely to suggest PsA than seropositive RA. Lesions in PIP, MCP, and wrist joints, as well as erosions, advanced bone damage, joint subluxations, dislocations, and joint space narrowing, were more common in seropositive RA patients than in seronegative RA patients. The ulnar styloid was more commonly affected in seronegative RA patients than in PsA patients. The study confirmed that types of bone lesions and their distribution in the hands and wrists can be useful in differentiating seropositive RA from PsA and suggests that seronegative RA varies in radiological presentation from seropositive RA and PsA.

## 1. Introduction

The hand and wrist are among the most common anatomical areas involved in rheumatic diseases. This is particularly true for rheumatoid arthritis (RA) and psoriatic arthritis (PsA) [1,2]. The hand and wrist are also involved in metabolic (e.g., gout) and endocrine (e.g., hyperparathyroidsm) diseases as well as in juvenile idiopathic arthritis, which is the most common rheumatic disease in juveniles. For decades, radiography (XR) has been a mainstay in imaging of inflammatory arthrtopathies, serving for diagnosing, monitoring the progression of disease, monitoring its complications and post-operative changes, and formulating prognosis [3,4,5,6]. Nowadays, ultrasound (US) and magnetic resonance imaging (MRI) enable earlier diagnosis; however, they frequently lack specificity, especially in the early stages, because all of the above-mentioned diseases can manifest with synovitis, tenosynovitis, and osteitis [7]. Radiographs remain a basic imaging technique used to detect and monitor bone damage and the disease progression [8].

***Radiographic pattern of hand and wrist involvement in seropositive rheumatoid arthritis, seronegative rheumatoid arthritis, and psoriatic arthritis.***

Seropositive RA is an autoimmune, inflammatory disease that is more frequent in women than in men, with a 4:1 ratio [9]. It is considered seropositive if either a rheumatoid factor (RF) or anti-citrullinated-protein antibodies (ACPA) are present in a patient’s serum [10]. Among RA patients, 60–80% test positive for ACPA [11] and approximately 80% test positive for RF [12].

Seropositive RA most typically affects small joints of the hands and feet—proximal interphalangeal (PIP), metacarpophalangeal (MCP), metatarsophalangeal (MTP), and wrist joints [13,14]. The involvement of distal interphalangeal (DIP) joints is rare [6].

The first radiographic signs of RA are periarticular soft tissue swelling and juxta-articular osteopenia, followed by joint space narrowing, subchondral cysts and bone erosions, and malalignments, as well as deformities, such as ‘Boutonniere’ deformity, ‘swan neck’, ‘Z thumb’, or ulnar deviation of fingers [15]. Late stages of disease can manifest with advanced bone destruction (e.g., osteolysis) as well as ankylosis (especially in wrist and carpometacarpal joints) [1]. Bone proliferations can occur in RA in the form of osteophytes, as development of secondary osteoarthritis (OA) by those patients is not uncommon [1].

**Figure 1:**
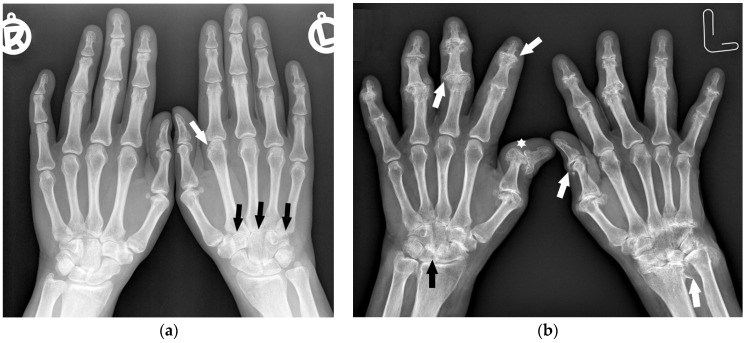
A 40-year-old female with early asymmetric seropositive rheumatoid arthritis (RA). (**a**) Posterior–anterior (PA) radiographs of bilateral hands show narrowing of the left radiocarpal joint (RCJ) and carpo-metatacarpal (CMC) joints (black arrows). Note the bone cyst in the left second metacarpal head and narrowing of the second metacarpophalangeal joint (MCP) (white arrow). (**b**) PA radiograph of the bilateral hands of the same patient, 13 years later, showed progression of the disease with increased juxta-articular osteoporosis. Note the erosive bone changes, advanced destructive changes, and joint narrowing in the RCJ and CMC joints. There are less advanced changes on the right side with joint space narrowing and bone cysts in the wrist (black arrow). Note proliferative osteoarthritic changes in left distal radioulnar joint (DRUJ) and bilateral first MCP and interphalangeal joints (white arrows). There is subluxation of the right thumb interphalangeal (IP) joint (star).

The distribution of involved joints in RA is widely described in the literature as “bilateral and symmetrical” [1,16]; however, the disease may begin as unilateral and asymmetric arthritis [15] (Figure 1).

Psoriatic arthritis has a more diverse musculoskeletal presentation. The classification by Moll and Wright distinguishes five main groups of PsA, of which three describe different patterns of hand and wrist involvement: polyarthritis, isolated involvement of the DIP joints in hands and feet, and psoriatic arthritis mutilans (PAM) [17].

Among these, isolated involvement of the DIP joints in hands is a hallmark of PsA, whereas PAM and polyarthritis can be indistinguishable from seropositive RA [15]. Laboratory tests may also be unreliable in the differentiation of PsA from seropositive RA. Although the lack of RF is suggestive of PsA [18], it can be found in up to 20% of PsA patients [19]. Moreover, ACPA antibodies are found in 20.9% of PsA patients according to Gruber et al. [20]. This study also found that, in the group of patients with PsA, those who were ACPA positive were more likely to present the polyarticular form of arthritis.

In 85% of patients, psoriatic skin manifestation precedes the development of PsA [12]. These findings and family history of psoriasis can suggest the diagnosis; however, at times, clinical data are unavailable to radiologists.

**Figure 2:**
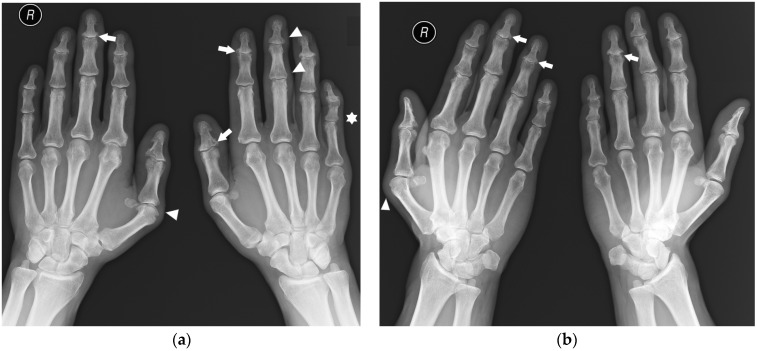
A 45-year-old male with psoriatic arthritis (PsA). PA (**a**) and Nørgaard views (**b**) of bilateral hands. Note the swan neck deformities, most prominent in the left small finger (star). There is soft tissue swelling around several distal interphalangeal (DIP) and thumb interphalangeal (IP) joints, as well as the right first MCP and left third proximal interphalangeal (PIP) joints (arrowheads). Note the scattered bone cysts, erosions, and proliferative bone changes at several DIP joints and the left thumb IP joint (arrows). There are erosions in the right first metacarpal (MC) head and a bone cyst and small erosion in right second MC head.

Erosions, bone cysts, and malalignments can be present in both PsA and RA. The hallmarks distinguishing hand and foot radiographs of PsA from RA are proliferative bone changes in combination with erosions, predilection to DIP joints, acroosteolysis (although rarely seen), lack of juxta-articular osteoporosis, and asymmetric distribution with the involvement of a single ray or several rays with dactylitis [1,6] (Figure 2). Advanced changes can form pencil-in-cup deformities [21].

**Figure 3:**
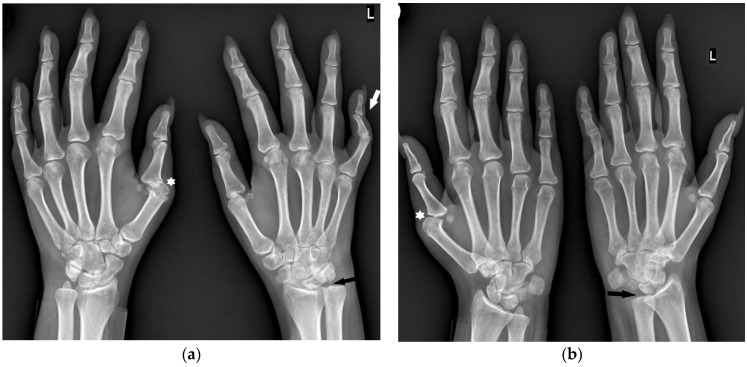
A 30-year-old female with seronegative RA. PA (**a**) and Nørgaard radiographs (**b**) of bilateral hands. Note the boutonnière deformities, most prominent in the left small finger (white arrow). There is left RCJ space narrowing with scattered bone cysts and chronic deformity of the scaphoid bone. Note the osteolysis of the left ulnar styloid process (black arrow). Scattered bone cysts are observed in the right carpal bones. Note the erosive changes and bone cysts in the right first MC head with subluxation of the MCP joint (star).

‘Seronegative RA’ is the least understood entity and only a few scientific studies describe the radiological image of this form. In a study by Gadelholt et al. [16], seronegative RA patients predominantly displayed carpal damage (Figure 3) with less-affected PIP joints and relative sparing of the feet compared with seropositive RA. Erosions and joint space narrowing were more prominent in seropositive RA, resulting in an overall higher disease activity score.

Radiographs of seronegative RA patients may, however, be diversified, because this is a heterogenous group with findings present in other diseases, including polyarticular OA, PsA, crystal arthropathies, and anti-synthetase syndrome [22]. Thus, the final diagnosis, at times, cannot be predicted in an early phase [23]. There are postulates of a common phenotype of seronegative, non-HLA-B27-associated, non-psoriatic arthritis [24].

As more disease-specific treatment has been introduced and bone damage in the aforementioned diseases can be delayed, the correct diagnosis at an early stage is currently paramount for the management of these diseases.

## 2. Aim of the Study

This paper aims to describe radiographic features in the hand and wrist in RA and PsA and the rarely-described seronegative RA in the era of modern therapy, taking into consideration the symmetry of involvement, affected compartments, and type of lesions. It also aims to identify the most differentiating radiographic characteristics of those diseases and compare them to those cited in the literature.

## 3. Materials and Methods

A retrospective analysis was performed on bilateral hand radiographs in three groups of patients routinely treated in a reference center for rheumatology in the years 2005–2020, identified using a clinical database. The groups consisted of 180 seropositive RA patients (29 males, 151 females, mean age at the point of acquisition of the analyzed radiographs of 53.4 y/o, SD 12.6), 154 PsA patients (45 males, 109 females, age median of 48.1 y/o, SD 12.4), and 36 seronegative RA patients (4 males, 32 females, age median of 53.1 y/o, SD 17.1). The patients were randomly selected from the clinical database and only those with a definitive, documented diagnosis and who presented at least one identifiable lesion on radiographs of both hands were included in the analysis. If more than one set of radiographs was available for a patient, the earliest set was chosen for the analysis. The sets of radiographs consisted of posterior–anterior (PA) and Nørgaard views of both hands that had been assessed by experienced radiologists. The demographic data are presented in Table 1.

At first, the radiographs were analyzed qualitatively for the appearance of typical features of inflammatory arthritis: juxta-articular osteoporosis, bone cysts, erosions, joint space narrowing, joint subluxations and dislocations, contracture ulnar deviation of fingers, proliferative bone changes, advanced bone destruction, ankylosis, and acroosteolysis.

Secondly, the affected compartments were identified on each radiograph, including DIP joints’ level, PIP joints’ level, thumb interphalangeal (IP) joint, MCP, carpometacarpal (CMC) joints, and carpal joints, as well as, separately, the styloid processes of the ulna and radius.

Then, the radiographs were analyzed for the symmetry of lesions with the consideration of three possible definitions of symmetry:

1. bilateral changes (lesions present in both hands);

2. changes affecting corresponding compartments (as defined above, e.g., some of DIP joints, not necessary the same in each hand, are affected in both hands);

3. ‘mirror-image’ symmetry (the same joints are affected in both hands).

Lastly, the three diseases were compared, i.e., seropositive RA versus PsA, seropositive RA versus seronegative RA, and seronegative RA versus PsA, taking into consideration the type of lesions, compartmental involvement, and symmetry of the lesions.

All radiographs were assessed by three experienced radiologists from the reference center of rheumatology and the final diagnosis was made by consensus.

**Table 1:** Demographic data of patients with seropositive rheumatoid arthritis (RA), seronegative RA, and psoriatic arthritis (PsA): sex and mean age at the acquisition of the radiographs.

	Seropositive RA	Seronegative RA	PsA
	*n* = 180	*n* = 36	*n* = 154
Female	151 (83.9%)	32 (88.9%)	109 (70.8%)
Male	29 (16.1%)	4 (11.1%)	45 (29.2%)
Mean age (SD)	53.4 (12.6)	53.1 (17.7)	48.1 (12.4)

## 4. Statistical Analysis

The frequency of individual changes in the groups of patients was compared using the chi-square test or the chi-square test with Yates’ correction; *p* < 0.05 was considered statistically significant. Diseases were differentiated in pairs: seropositive RA versus PsA, seropositive RA versus seronegative RA, and seronegative RA versus PsA.

Univariable and multivariable logistic regression were used to indicate factors differentiating two diseases. Multivariate models were created from variables that were statistically significant in the univariate analysis. In each differentiation, three multivariable models were created, which included age; sex; and type of symmetry, location of lesions, and types of lesions.

An odds ratio (OR) with a 95% confidence interval was calculated for each variable.

Factors that occur in 100% or 0% of patients of the analyzed group (e.g., acroosteolysis, which was not observed in any seronegative RA patients) cannot be assessed in logistic regression; however, they can be suggestive of the diagnosis.

## 5. Results

The results feature the general overview of the findings (Section 5.1) followed by the outcomes of the comparative analysis in pairs: seropositive RA versus PsA (Section 5.2), seropositive RA versus seronegative RA (Section 5.3), and seronegative RA versus PsA (Section 5.4).

### 5.1. Overview of the Symmetry, Distribution, and Radiographic Lesions in the Three Compared Diseases

**Figure 4:**
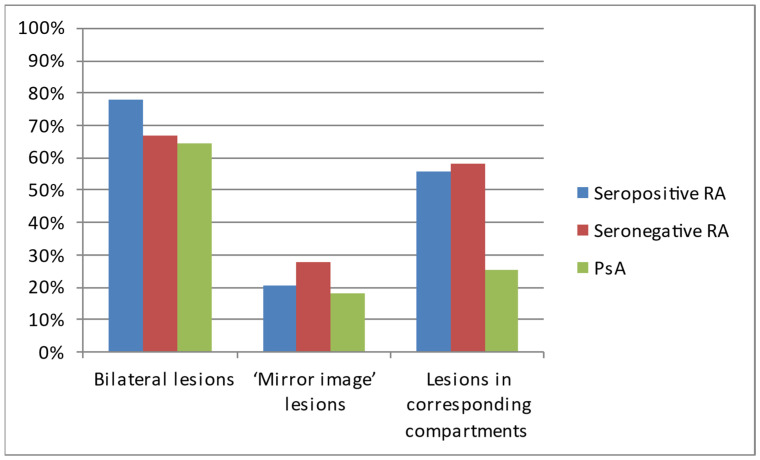
The occurrence of symmetric lesions on hand radiographs of analyzed patients with seropositive RA, seronegative RA, and PsA.

Regarding the pattern of hands’ involvement, bilateral lesions were observed in most of the patients with each of the analyzed diseases (Table 2, Figure 4).

**Figure 5:**
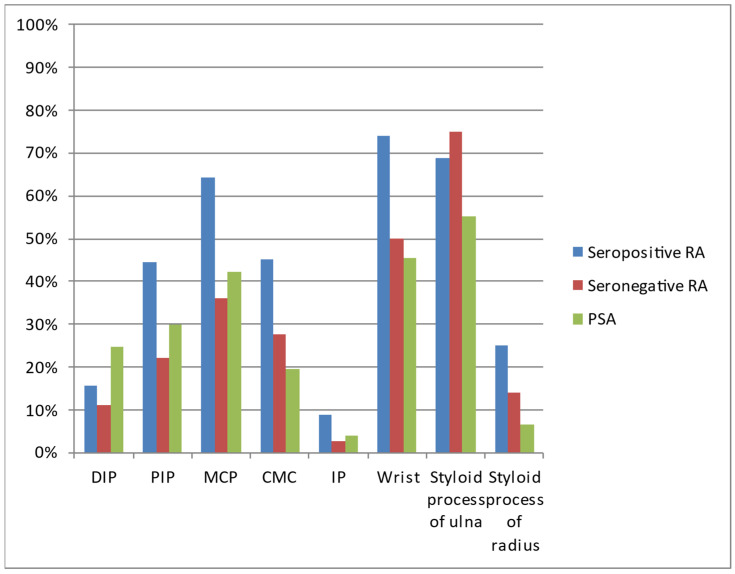
Locations of lesions on hand radiographs of analyzed patients with seropositive RA, seronegative RA, and PsA.

The most common locations in all three diseases were the wrist, styloid process of the ulna, and MCP joints (Table 3, Figure 5). All three diseases affected the DIP joints in varied percentages.

**Figure 6:**
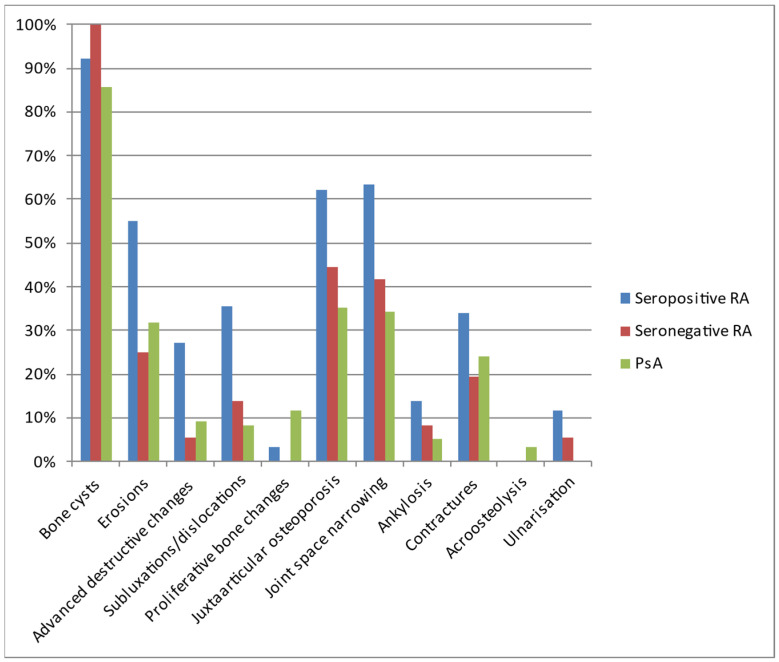
Types of lesions observed on hand radiographs of analyzed patients with seropositive RA, seronegative RA, and PsA.

The most commonly diagnosed lesions included bone cysts, followed by joint space narrowing, juxta-articular osteoporosis, and erosions, although the exact percentage of occurrence varied between the diseases (Table 4, Figure 6). In general, inflammatory/destructive changes prevailed in patients with seropositive RA. They had no acroosteolysis and no bone proliferations. The latter were the only pathology found more often in PsA. In contrast, no ulnar deviation was found in PsA. The most common location of seronegative RA was the styloid process of the ulna, followed by wrist and MCP joints.

### 5.2. Differentiating Seropositive RA from PsA

Table 4 presents the considered types of symmetric involvement in the three analyzed groups of patients (Table 5). It turned out that, if symmetry is considered as changes affecting corresponding compartments of both hands, the patients with symmetrical changes were diagnosed with seropositive RA three times more often than those with PsA. Other considered types of symmetry failed to provide statistically significant differentiation.

In the model analyzing the location of lesions, we showed that lesions affecting the PIP joints, wrist, or styloid process of the radius are about twice as common in patients with seropositive RA compared with those with PsA (Table 6). On the other hand, DIP joints were more than twice as likely to be affected in PsA compared with seropositive RA patients.

In the model with lesion types, it was found that the chance of joint subluxation or dislocation was almost three times greater in patients with seropositive RA compared with patients with PsA (Table 7). Juxta-articular osteoporosis and joint space narrowing were also suggestive of seropositive RA. In contrast, proliferative bone changes were five times more likely in PsA than in seropositive RA (Table 7).

### 5.3. Differentiating Seropositive RA from Seronegative RA

The types of symmetry failed to provide a statistically significant differentiation between seropositive and seronegative RA (Table 8).

In the model analyzing the location of lesions, univariate analysis suggested that patients with changes at the PIP, MCP, and wrist joints are approximately three times as likely to be diagnosed with seropositive RA than seronegative RA (Table 9). However, in multivariate analysis, this was not statistically relevant. Further research would prove useful to confirm these findings.

For the lesion types, univariate analysis showed that erosions, advanced destructive changes, joint subluxations and dislocations, and joint space narrowing were more suggestive of seropositive RA than seronegative RA (Table 10), but this was not confirmed as statistically significant in multivariate analysis.

### 5.4. Differentiating Seronegative RA from PsA

An analysis of the type of symmetry showed that the symmetry of disease in corresponding compartments of both hands increased the chance of patients being diagnosed with seronegative RA by approximately three times compared with those with PsA (Table 11). Other considered types of symmetry failed to provide a statistically significant differentiation.

In the model analyzing the location of lesions, the affected styloid process of the ulna is more suggestive of seronegative RA than PsA (Table 12).

Finally, the lesion type analysis did not prove useful for differentiating seronegative RA from PsA (Table 13).

## 6. Discussion

**Figure 7:**
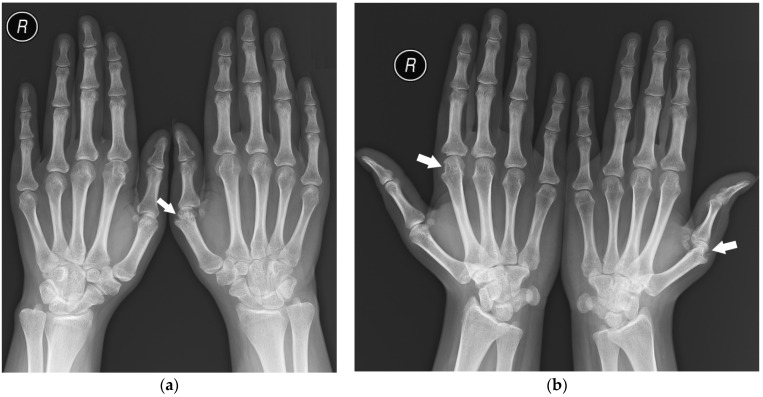
A 25-year-old female with seropositive RA. PA (**a**) and Nørgaard radiographs (**b**) of bilateral hands. Erosions, juxta-articular osteoporosis, joint space narrowing, and soft tissue swelling in several MCP joints, especially the left first MCP and right second MCP joints (arrows). Note the changes affecting the same compartments.

The analysis showed that seropositive and seronegative RA manifest symmetric involvement of corresponding compartments of both hands (Figure 7) more often than PsA, which is consistent with the literature [1,6,16]. The other considered types of symmetry were not proved to be statistically relevant.

As described in the literature [1,6], the hallmarks differentiating PsA from seropositive RA are erosions with concomitant proliferative bone changes at the DIP joints. On the other hand, lesions in PIP joints, the wrist, or the styloid process of the radius, as well as the presence of joint subluxation/dislocation, juxta-articular osteoporosis, and joint space narrowing, suggest seropositive RA over PsA.

Acroosteolysis was observed exclusively in a small group of patients with PsA (3.3%); therefore, it could not be included in statistical analysis. It could be considered a highly specific, although rare, feature of PsA, as mentioned in the literature [1]. Additionally, it should be pointed that our study included the earliest available hand radiographs of the patients, while acroosteolysis may be more common in later stages of the disease [15].

Similarly, ulnar deviation of the fingers was observed in patients with seropositive RA (11.7%) and seronegative RA (5.6%), but it was not described in PsA patients and was not suited for logistic regression. However, it points against the diagnosis of PsA. Indeed, this feature has not been listed among PsA-specific lesions [1,9,15].

Regarding the location of the lesions, the involvement of the styloid process of the ulna was the most differentiating feature between seronegative RA and PsA, with the predilection to seronegative RA, which is not mentioned in the literature.

**Figure 8:**
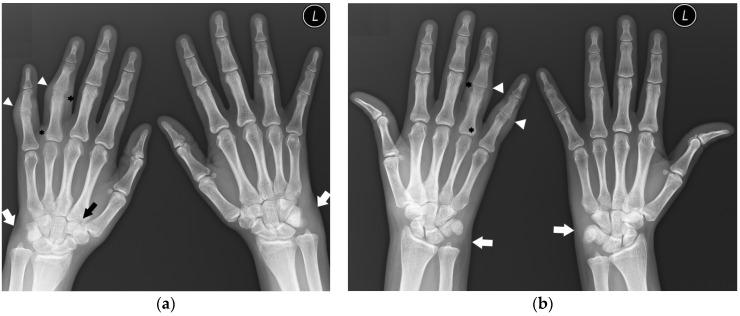
A 31-year-old female with PsA. PA (**a**) and Nørgaard radiographs (**b**) of bilateral hands. Note the sparring of the DIP joints. There is soft tissue swelling and contracture at the level of the fourth and fifth digits on the PIP joints of the right hand (arrowheads). Note the soft tissue swelling around both wrists, especially on the ulnar side (white arrows). There are erosions at the base and head of the right fourth digit proximal phalanx with discrete proliferative bone changes (stars). Note the narrowing of the left fourth PIP and right second CMC joint spaces with bone cysts at the base of the right second MC bone (black arrow).

In our study, in 72% (111) of PsA patients, the DIP joints were not involved (Figure 8), without evidence of proliferative bone changes. This indicates the lack of the main hallmark of PsA, consisting of DIP involvement with concomitant proliferative and erosive changes. Therefore, these findings can easily mimic other rheumatic diseases, especially in the early stages of the disease, as mentioned by Sudoł-Szopińska et al. [15].

In our analysis, lesions in PIP, MCP, and wrist joints were more common in seropositive RA than in seronegative RA, unlike in the study by Gadeholt et al. [16], in which seronegative patients predominantly displayed carpal damage. However, their study centered on the relative occurrence of changes in wrists compared with other sites and pointed to an overall lower disease activity score in seronegative patients. Therefore, the results of our study and those of their study may not contradict each other. Further studies would be needed to verify this.

In the model with lesion types, univariate analysis showed that erosions, advanced destructive changes, joint subluxations and dislocations, and joint narrowing are more suggestive of seropositive RA than seronegative RA, but this was not confirmed as statistically significant in multivariate analysis and further studies are needed.

None of our patients with seronegative RA had proliferative bone changes described on hand radiographs. Although it cannot be assessed in the statistical analysis, our study indicates that proliferative bone changes point against the diagnosis of seronegative RA. Further studies are needed for statistically reliable data.

Finally, it should be mentioned that, in the early stages of the analyzed diseases, radiography is frequently negative and MRI and musculoskeletal ultrasound play a key role in detecting soft tissue changes, which is necessary to slow or stop the disease progression before irreversible bone damage occurs [25,26].

## 7. Limitations

The patients were included in the study based on their International Classification of Diseases 10th Revision (ICD-10) code in the clinical database. The study included patients treated in the years 2005–2020. During this period, the classification criteria for the diagnosis of inflammatory rheumatic diseases were changed by international committees. Most importantly, the new classification criteria for RA by EULAR/ACR that were introduced in 2010 [27] do not require positive radiographic findings to make the diagnosis.

It is possible that some of the patients had other coexisting diseases. In particular, contractures and malalignments may have several causes (e.g., post-traumatic) that are indistinguishable on radiographs without clinical data.

The duration of the disease for each patient was unavailable to the researchers and thus could not be included in the analysis.

The analysis was based on subjective assessment of the radiographs by three radiologists with decades of experience. However, early, subtle changes may at times be elusive and ambiguous without using other advanced imaging methods.

The small group of patients with seronegative RA made it impossible to obtain significant logistic regression results in differentiating it from seropositive RA and PsA.

## 8. Conclusions

The hand and wrist are frequently involved in several rheumatic diseases. At their early stages, radiographs may be negative or show subtle lesions that prove challenging to assign to a specific disease.

Rheumatoid arthritis is commonly described in the literature as a bilateral, symmetric disease. The analysis of hand radiographs in our study clarified that symmetric distribution of the lesions should be understood as ‘lesions present in corresponding compartments’. Such symmetry is indeed more suggestive of seropositive or seronegative RA than of PsA.

This study confirmed that the type of lesions and their distribution can be useful in differentiating seropositive RA (involvement of the PIP joints, wrist, and styloid process of the radius, as well as subluxations/dislocations, juxta-articular osteoporosis, and joint space narrowing) from PsA (involvement of the DIP joints and proliferative bone changes).

Seronegative RA varies in radiological presentation from seropositive RA and PsA, considering both the distribution and type of lesions. However, further studies are needed for statistically relevant data. The authors hope that this work will be helpful in diagnosing patients with RA and PsA, especially in settings without typical serological findings and with unknown clinical data.

## Figures and Tables

**Table 2:** The occurrence of symmetric lesions on hand radiographs of analyzed patients with seropositive RA, seronegative RA, and PsA.

Type of Symmetry	Seropositive RA *n* = 180	Seronegative RA *n* = 36	PsA *n* = 154
Bilateral lesions	140 (77.8%)	24 (66.7%)	99 (64.3%)
‘Mirror-image’ lesions	37 (20.6%)	10 (27.8%)	28 (18.2%)
Lesions in corresponding compartments	100 (55.6%)	21 (58.3%)	39 (25.3%)

**Table 3:** Locations of lesions on hand radiographs of analyzed patients with seropositive RA, seronegative RA, and PsA.

Location	Seropositive RA *n* = 180	Seronegative RA *n* = 36	PSA *n* = 154
Distal interphalangeal joints (DIP)	28 (15.6%)	4 (11.1%)	38 (24.7%)
Proximal interphalangeal joints (PIP)	80 (44.4%)	8 (22.2%)	46 (29.9%)
Metacarpophalangeal joints (MCP)	116 (64.4%)	13 (36.1%)	65 (42.2%)
Carpometacarpal joints (CMC)	81 (45.0%)	10 (27.8%)	30 (19.5%)
Interphalangeal joint of the thumb (IP)	16 (8.9%)	1 (2.8%)	6 (3.9%)
Wrist	133 (73.9%)	18 (50.0%)	70 (45.5%)
Styloid process of the ulna	124 (68.9%)	27 (75.0%)	85 (55.2%)
Styloid process of the radius	45 (25.0%)	5 (13.9%)	10 (6.5%)

**Table 4:** Types of lesions observed on hand radiographs of analyzed patients with seropositive RA, seronegative RA, and PsA.

Lesion	Seropositive RA *n* = 180	Seronegative RA *n* = 36	PsA *n* = 154
Bone cysts	166 (92.2%)	36 (100.0%)	132 (85.7%)
Erosions	99 (55.0%)	9 (25.0%)	49 (31.8%)
Advanced destructive changes	49 (27.2%)	2 (5.6%)	14 (9.1%)
Subluxations/dislocations	64 (35.6%)	5 (13.9%)	13 (8.4%)
Proliferative bone changes	6 (3.3%)	0 (0.0%)	18 (11.7%)
Juxta-articular osteoporosis	112 (62.2%)	16 (44.4%)	54 (35.1%)
Joint space narrowing	114 (63.3%)	15 (41.7%)	53 (34.4%)
Ankylosis	25 (13.9%)	3 (8.3%)	8 (5.2%)
Contractures	61 (33.9%)	7 (19.4%)	37 (24.0%)
Acroosteolysis	0 (0.0%)	0 (0.0%)	5 (3.3%)
Ulnarisation	21 (11.7%)	2 (5.6%)	0 (0.0%)

**Table 5:** Logistic regression for the dependent variable of seropositive RA vs. the independent variable of PsA considering the types of symmetry. Variables that were significant in the univariate analysis were included in the multivariate model.

Factor	Univariate Analysis		Multivariate Analysis	
	OR (95% CI)	*p*	OR (95% CI)	*p*
Female sex	**2.15 (1.27–3.64)**	**0.005**	**1.88 (1.06–3.31)**	**0.030**
Age (increase by 1 year)	**1.03 (1.02–1.05)**	**<0.001**	**1.03 (1.01–1.05)**	**0.001**
Bilateral lesions	**1.94 (1.20–3.15)**	**0.007**	0.87 (0.48–0.57)	0.638
‘Mirror-image’ lesions	1.16 (0.67–2.01)	0.585	-	-
Lesions in corresponding compartments	**3.67 (2.31–5.88)**	**<0.001**	**3.73 (2.13–6.53)**	**<0.001**

**Table 6:** Logistic regression for the dependent variable of seropositive RA vs. the independent variable of PsA considering the location of the lesions.

Factor	Univariate Analysis		Multivariate Analysis	
	OR (95% CI)	*p*	OR (95% CI)	*p*
Female sex	**2.15 (1.27–3.64)**	**0.005**	**1.81 (1.00–3.28)**	**0.049**
Age (increase by 1 year)	**1.03 (1.02–1.05)**	**<0.001**	**1.03 (1.01–1.05)**	**0.015**
DIP	**0.56 (0.41–0.97)**	**0.038**	**0.38 (0.20–0.74)**	**0.005**
PIP	**1.88 (1.19–2.96)**	**0.006**	**2.20 (1.27–3.81)**	**0.005**
MCP	**2.48 (1.60–3.86)**	**<0.001**	1.68 (0.98–2.89)	0.061
CMC	**3.38 (2.06–5.55)**	**<0.001**	1.46 (0.80–2.66)	0.223
IP	**2.41 (0.92–6.31)**	0.074	-	-
Wrist	**3.40 (2.15–5.38)**	**<0.001**	**1.94 (1.12–3.38)**	**0.024**
Styloid process of the ulna	**1.80 (1.15–2.81)**	**0.010**	1.47 (0.88–2.46)	0.141
Styloid process of the radius	**4.80 (2.33–9.90)**	**<0.001**	**2.58 (1.13–5.89)**	**0.015**

**Table 7:** Logistic regression for the dependent variable of seropositive RA vs. the independent variable of PsA considering the types of lesions.

Factor	Univariate Analysis		Multivariate Analysis	
	OR (95% CI)	*p*	OR (95% CI)	*p*
Female sex	**2.14 (1.33–3.45)**	**0.002**	-	-
Age (increase by 1 year)	**1.04 (1.02–1.05)**	**<0.001**	**1.03 (1.01–1.05)**	**0.004**
Bone cysts	1.98 (0.97–4.01)	0.059	-	-
Erosions	**2.62 (1.67–4.10)**	**<0.001**	1.40 (0.72–2.75)	0.324
Advanced destructive changes	**3.74 (1.97–7.09)**	**<0.001**	1.03 (0.41–2.56)	0.953
Subluxations/dislocations	**5.98 (3.14–11.40)**	**<0.001**	**2.83 (1.29–6.21)**	**0.010**
Proliferative bone changes	**0.26 (0.10–0.67)**	**0.006**	**0.21 (0.07–0.62)**	**0.005**
Juxta-articular osteoporosis	**3.05 (1.95–4.77)**	**<0.001**	**1.96 (1.13–3.40)**	**0.017**
Joint space narrowing	**3.29 (2.10–5.16)**	**<0.001**	**1.91 (1.04–3.52)**	**0.038**
Ankylosis	**2.94 (1.29–6.73)**	**0.003**	1.24 (0.43–3.61)	0.690
Contractures	**1.62 (1.00–2.62)**	**0.049**	1.23 (0.70–2.16)	0.476
Acroosteolysis	-	0.998	-	-
Ulnarisation	-	0.997	-	-

**Table 8:** Logistic regression for the dependent variable of seropositive RA vs. the independent variable of seronegative RA considering the types of symmetry.

Factor	Univariate Analysis		Multivariate Analysis	
	OR (95% CI)	*p*	OR (95% CI)	*p*
Female sex	0.65 (0.21–1.98)	0.449	-	-
Age (increase by 1 year)	1.00 (0.98–1.03)	0.903	-	-
Bilateral lesions	1.75 (0.81–3.81)	0.158	-	-
‘Mirror-image’ lesions	0.67 (0.30–1.52)	0.340	-	-
Lesions in corresponding compartments	0.89 (0.43–1.84)	0.759	-	-

**Table 9:** Logistic regression for the dependent variable of seropositive RA vs. the independent variable of seronegative RA considering the location of the lesions.

Factor	Univariate Analysis		Multivariate Analysis	
	OR (95% CI)	*p*	OR (95% CI)	*p*
Female sex	0.65 (0.21–1.98)	0.449	-	-
Age (increase by 1 year)	1.00 (0.98–1.03)	0.903	-	-
DIP	1.47 (0.48–4.49)	0.319	-	-
PIP	**2.80 (1.21–6.48)**	**0.016**	2.29 (0.96–5.48)	0.062
MCP	**3.21 (1.52–6.76)**	**0.002**	2.01 (0.86–4.72)	0.107
CMC	2.13 (0.97–4.67)	0.060	-	
IP	3.42 (0.44–26.61)	0.241	-	-
Wrist	**2.83 (1.36–5.89)**	**0.005**	1.96 (0.85–4.49)	0.113
Styloid process of the ulna	0.74 (0.33–1.67)	0.467	-	-
Styloid process of the radius	2.07 (0.76–5.64)	0.156	-	-

**Table 10:** Logistic regression for the dependent variable of seropositive RA vs. the independent variable of seronegative RA considering the types of lesions.

Factor	Univariate Analysis		Multivariate Analysis	
	OR (95% CI)	*p*	OR (95% CI)	*p*
Female sex	0.65 (0.21–1.98)	0.449	-	-
Age (increase by 1 year)	1.00 (0.98–1.03)	0.903	-	-
Bone cysts	-	-	-	-
Erosions	**3.67 (1.63–8.24)**	**0.002**	2.01 (0.76–5.33)	0.160
Advanced destructive changes	**6.34 (1.47–27.47)**	**0.013**	2.76 (0.52–14.71)	0.236
Subluxations/dislocations	**3.42 (1.27–9.23)**	**0.015**	1.64 (0.53–5.12)	0.391
Proliferative bone changes	-	-	-	-
Juxta-articular osteoporosis	2.06 (1.00–4.24)	0.050	-	
Joint space narrowing	**2.42 (1.17–5.01)**	**0.018**	1.17 (0.50–2.73)	0.722
Ankylosis	1.77 (0.51–6.22)	0.371	-	-
Contractures	2.12 (0.88–5.13)	0.094	-	-
Acroosteolysis	-	-	-	-
Ulnarisation	2.25 (0.50–10.03)	0.290	-	-

**Table 11:** Logistic regression for the dependent variable of seronegative RA vs. the independent variable of PsA considering the types of symmetry.

Factor	Univariate Analysis		Multivariate Analysis	
	OR (95% CI)	*p*	OR (95% CI)	*p*
Female sex	**3.30 (1.10–9.88)**	**0.033**	2.55 (0.83–7.85)	0.104
Age (increase by 1 year)	1.03 (1.00–1.06)	0.050	-	-
Bilateral lesions	1.11 (0.52–2.39)	0.788	-	-
‘Mirror-image’ lesions	1.73 (0.75–3.99)	0.199	-	-
Lesions in corresponding compartments	**4.13 (1.94–8.79)**	**<0.001**	**3.50 (1.62–7.57)**	**0.001**

**Table 12:** Logistic regression for the dependent variable of seronegative RA vs. the independent variable of PsA considering the location of the lesions.

Factor	Univariate Analysis		Multivariate Analysis	
	OR (95% CI)	*p*	OR (95% CI)	*p*
Female sex	**3.30 (1.10–9.88)**	**0.033**	**3.32 (1.10–10.00)**	**0.033**
Age (increase by 1 year)	1.03 (1.00–1.06)	0.050	-	-
DIP	0.38 (0.13–1.15)	0.087	-	-
PIP	0.67 (0.28–1.58)	0.362	-	
MCP	0.77 (0.37–1.64)	0.504	-	-
CMC	1.59 (0.69–3.65)	0.274	-	
IP	0.71 (0.08–6.04)	0.750	-	-
Wrist	1.20 (0.58–2.48)	0.623	-	-
Styloid process of the ulna	**2.44 (1.07–5.52)**	**0.033**	**2.44 (1.07–5.59)**	**0.034**
Styloid process of the radius	2.32 (0.74–7.27)	0.148	-	-

**Table 13:** Logistic regression for the dependent variable of seronegative RA vs. the independent variable of PsA considering the types of lesions.

Factor	Univariate Analysis		Multivariate Analysis	
	OR (95% CI)	*p*	OR (95% CI)	*p*
Female sex	**3.30 (1.10–9.88)**	**0.033**	-	-
Age (increase by 1 year)	1.03 (1.00–1.06)	0.050	-	-
Bone cysts	-	-	-	-
Erosions	0.71 (0.31–1.63)	0.425	-	
Advanced destructive changes	0.59 (0.13–2.71)	0.496	-	-
Subluxations/dislocations	1.75 (0.58–5.27)	0.320	-	-
Proliferative bone changes	-	-	-	-
Juxta-articular osteoporosis	1.48 (0.71–3.09)	0.295	-	
Joint space narrowing	1.36 (0.65–2.86)	0.455	-	-
Ankylosis	1.66 (0.42–6.59)	0.472	-	-
Contractures	0.76 (0.31–1.89)	0.558	-	-
Acroosteolysis	-	-	-	-
Ulnarisation	-	-	-	-

## Data Availability

Data is unavailable due to privacy.
